# Intelligent Fault Diagnosis of Diesel Engines via Extreme Gradient Boosting and High-Accuracy Time–Frequency Information of Vibration Signals

**DOI:** 10.3390/s19153280

**Published:** 2019-07-25

**Authors:** Jianfeng Tao, Chengjin Qin, Weixing Li, Chengliang Liu

**Affiliations:** State Key Laboratory of Mechanical System and Vibration, School of Mechanical Engineering, Shanghai Jiao Tong University, Shanghai 200240, China

**Keywords:** diesel engine, extreme gradient boosting-based misfire diagnosis method, high-accuracy time–frequency information, vibration signal, feature dimensionality reduction

## Abstract

Accurate and timely misfire fault diagnosis is of vital significance for diesel engines. However, existing algorithms are prone to fall into model over-fitting and adopt low energy-concentrated features. This paper presents a novel extreme gradient boosting-based misfire fault diagnosis approach utilizing the high-accuracy time–frequency information of vibration signals. First, diesel engine misfire tests were conducted under different spindle speeds, and the corresponding vibration signals were acquired via a triaxial accelerometer. The time-domain features of signals were extracted by using a time-domain statistics method, while the high-accuracy time–frequency domain features were obtained via the high-resolution multisynchrosqueezing transform. Thereafter, considering the nonlinearity and high dimensionality of the original characteristic data sets, the locally linear embedding method was employed for feature dimensionality reduction. Eventually, to avoid model overfitting, the extreme gradient boosting algorithm was utilized for diesel engine misfire fault diagnosis. Experiments under different spindle speeds and comprehensive comparisons with other evaluation methods were conducted to demonstrate the effectiveness of the proposed extreme gradient boosting-based misfire diagnosis method. The results verify that the highest classification accuracy of the proposed extreme gradient boosting-based algorithm is up to 99.93%. Simultaneously, the classification accuracy of the presented approach is approximately 24.63% higher on average than those of algorithms that use wavelet packet-based features. Moreover, it is shown that it obtains the minimum root mean squared error and can effectively prevent the model from falling into overfitting.

## 1. Introduction

Due to their high reliability and thermal efficiency, low cost, and long useful life, diesel engines have been vastly utilized in trucks and certain private vehicles [1]. However, caused by the irreversible machine aging process, the failure of components, and harsh working environments, various sorts of faults will frequently occur. Among them, cylinder misfire is a type of commonly seen fault. The occurrence of misfire will result in low output torque and inadequate power, and even cause severe damage to the machine equipment. In addition, it will also lead to excessive fuel consumption and terrible air pollution [2,3,4]. For these reasons, it is becoming increasingly important to achieve an accurate and timely fault diagnosis of diesel engine misfire, and corresponding research has gained much attention from both academia and industry.

Over the past decades, fault diagnosis and prognosis have gained increasing research attention because of the ever-increasing demand for the reliability and safety of the mechanical equipment in modern industry and industrial systems [5,6,7]. Generally, they can be categorized into four classes, i.e., model-based methods, data-driven methods, knowledge-based methods, and hybrid fault methods. For example, Nguyen and co-workers [8] proposed a data-driven percentile measure-based prognostic method for batch manufacturing processes. By combining physical knowledge and data analysis, Benmoussa et al. [9] developed a hybrid approach for estimation of the remaining useful life, in which no prior knowledge of the degradation process was needed. For the case of misfire fault diagnosis, it can be divided into two categories, i.e., the model-based methods and the data-driven methods. The model-based approaches usually include data generated from simulated models under nominal and fault conditions, and also require an adequate knowledge of an ordinarily dynamic process model in the form of a mathematical structure and parameters [10,11]. For instance, Jung et al. [12] presented a computationally simple model-based misfire detection algorithm, in which an automatic tuning method was used for the training of data. Wang and Chu [13] employed the mean deviation torque during the power stroke derived from the estimated deviation torque to detect engine misfires. To estimate the combustion torque from angular velocity measurement, Kiencke [14] proposed a simplified engine model based on the Kalman filter. However, due to the complex dynamics and kinematics characteristics, it is quite complicated to establish accurate theoretical models for engine cylinder misfire faults, making the model-based diagnosis methods less practical [15]. Fortunately, the data-driven methods provide a more practical solution for the misfire fault diagnosis of diesel engines. With the considerable development of computational ability and pattern recognition theory, the data-driven methods can achieve more satisfactory diagnostic results. Singh and co-workers [16] presented an improved engine misfire detection method via sound quality metrics of radiated sound and a support vector machine (SVM) classifier. Liu et al. [17] proposed an effective misfire detection method for a turbocharged diesel engine based on an artificial neural network model. To detect misfire fault by comparing data with the actual crankshaft speed, Chen et al. [18] adopted the extended neural network based on regression theory to calculate the crankshaft speed. Based on a multi-layer perceptron and probabilistic neural network, Chen and co-workers [19] proposed an optimized misfire detection algorithm to identify the location and degree of various misfire faults. By extracting crucial features from original vibrational signals, Sharma et al. [20] utilized multiple decision tree algorithms to establish the optimal misfire fault detection tree. Wu and co-workers [21] employed wavelet packet transform and artificial neural network techniques to build a fault diagnostic system for internal combustion engines. Hu et al. [22] proposed a multivariate statistical analysis method for misfire detection, in which a statistical characteristic analysis of crankshaft speed signals was conducted. Szabo and Bakucz [23] applied the fuzzy deep learning algorithm to identify the misfire characteristics and classify vibration signals acquired from different misfire working conditions. Babu and co-workers [24] applied multiple machine learning approaches to diagnose misfire faults based on the vibration signal.

However, in-depth analysis has revealed that current data-driven methods for misfire fault diagnosis mainly utilize spectrum analysis, traditional wavelet decomposition, wavelet packet decomposition, and empirical mode decomposition to extract significant features [15,16,17,18,19,20,21,22,23,24,25,26,27]. Restricted by the Heisenberg uncertainty principle, the classical processing methods suffer from a relatively low time–frequency resolution. Therefore, they are not competent enough to accurately characterize the nonstationary behaviors of the measured signals. Meanwhile, artificial neural networks and complex identification models are prone to falling into local extremum and overfitting for the actual engine misfire detection. For these reasons, the existing diagnostic algorithms face the challenges of easy over-fitting and a low feature extraction accuracy. To address these problems and achieve an accurate and timely fault diagnosis of diesel engine misfire, this paper develops a novel diagnosis method by combing the multisynchrosqueezing transform (MSST), locally linear embedding (LLE), and extreme gradient boosting, in which the advantages of all sides are preserved. To overcome the intrinsic weakness of the characteristic obtained with the traditional time–frequency analysis approach, a hybrid feature extraction method that combines time-domain statistical characteristic parameters and MSST-based feature extraction is proposed to extract the most sensitive and slightest signal features. Subsequently, LLE is embedded into the feature extraction process to eliminate redundant information hidden in original features. Eventually, to prevent the model falling into overfitting, the extreme gradient boosting algorithm is utilized to construct an engine misfire fault classification evaluator.

The remainder of this paper is organized as follows. In Section 2, the diagnosis scheme of misfire detection based on the vibration signal and related theories of MSST and extreme gradient boosting are presented. Meanwhile, the specific procedures of the proposed approach are also described in detail. Subsequently, the experimental setup and specific diesel engine rig tests with different spindle speeds and different misfire fault types are presented in Section 3. Thereafter, in Section 4, the performance of the proposed extreme gradient boosting-based algorithm is illustrated and verified by comparing it with several evaluation methods. Finally, Section 5 gives the concluding remarks.

## 2. The Proposed Extreme Gradient Boosting-Based Diagnosis Method

As presented in the literature, when a misfire fault occurs in the diesel engine, the vibration state of the cylinder head is obviously different from that under a normal working condition, which indicates that vibration signals of the cylinder head under different misfire working conditions contain corresponding fault characteristic indicators. Therefore, proper signal processing methods can be utilized to extract features from vibration signals under various misfire conditions and further construct data sets. On this basis, effective pattern recognition methods are applied to train and test the feature data sets, and the classification evaluation of each sample set is then completed.

**Figure 1:**
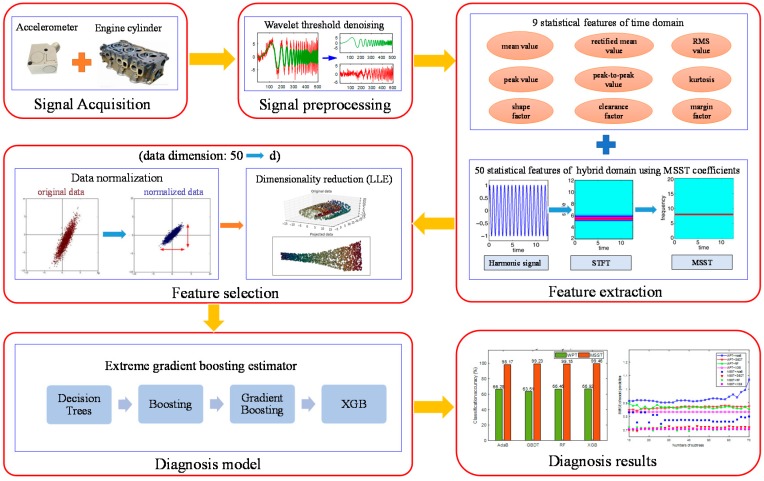
The proposed flow chart of diesel engine misfire diagnosis.

According to the above diagnosis scheme, the performance of misfire fault diagnosis can be improved by the following two aspects, i.e., extracting more accurate and sensitive features from the original vibration signals, and selecting a classifier with a strong generalization ability and high efficiency. Consequently, this paper develops a novel approach combining the multisynchrosqueezing transform and extreme gradient boosting for diesel engine misfire faults, in which the problem of a low resolution in feature extraction and model overfitting in the classification of engine misfire fault diagnosis can be effectively overcome. The flow chart of the proposed extreme gradient boosting-based misfire diagnosis method is presented in Figure 1.

In the first step, vibration signals of engine cylinder heads are acquired through experiments for testing the performance of misfire in engines with different spindle speeds. After signal denoising with the wavelet threshold method, features of signals are extracted by using a time-domain statistics method and the MSST algorithm. Then, the locally linear embedding method is adopted for effective feature dimensionality reduction. The extreme gradient boosting algorithm is finally utilized to construct an efficient and anti-overfitting evaluator. To better illustrate the proposed method, two crucial algorithms, i.e., the multisynchrosqueezing transform and extreme gradient boosting, are introduced briefly as follows.

### 2.1. Multisynchrosqueezing Transform

Similar to the synchrosqueezing transform (SST) [28,29], the MSST also belongs to the post-processing tool of conventional time–frequency analysis methods; for instance, the short-time Fourier transform (STFT). However, with the aid of a novel iterative procedure, it can effectively improve the time–frequency resolution of SST when addressing time-varying signals, and simultaneously presents a perfect signal reconstruction [30].

Now, the MSST-based feature extraction process is illustrated with a time-domain signal *s*(*t*) as an example. For clarity, we begin with SST based on an STFT framework. Additionally, the STFT of the time-domain signal *s*(*t*) is represented as

(1) $$F_{s}(t,\omega )=\int_{-\infty }^{\infty }g(u-t)s(u)e^{-i\omega \left(u-t\right)}du$$

where *g* denotes the compactly supported window function.

The SST employs a frequency reallocation operator to gather all STFT coefficients with the same instantaneous frequency, which is expressed as

(2) $$Ts(t,\eta )=\int_{-\infty }^{+\infty }F_{s}(t,\omega )\delta (\eta -\hat{\omega }(t,\omega ))d\omega$$

in which *δ* represents the Dirac function and $\hat{\omega }(t,\omega )$ denotes the instantaneous frequency for the STFT result.

For MSST, it repeatedly applies a new SST operation to the already acquired SST result. Through multiple iterations of SST, the energy of the time–frequency representation can be concentrated in a step-wise manner. Denoted by the iteration times *n_i_* (*n_i_* ≥ 2), the MSST can be formulated as

(3) $$\begin{array}{c} Ts^{\left[2\right]}\left(t,\eta \right)=\int_{-\infty }^{+\infty }Ts^{\left[1\right]}(t,\omega )\delta (\eta -\hat{\omega }(t,\omega ))d\omega \\ Ts^{\left[3\right]}\left(t,\eta \right)=\int_{-\infty }^{+\infty }Ts^{\left[2\right]}(t,\omega )\delta (\eta -\hat{\omega }(t,\omega ))d\omega \\ \vdots \\ Ts^{\left[n_{i}\right]}\left(t,\eta \right)=\int_{-\infty }^{+\infty }Ts^{\left[n_{i}-1\right]}(t,\omega )\delta (\eta -\hat{\omega }(t,\omega ))d\omega \end{array}$$

where $Ts^{\left[1\right]}(t,\omega )$ represents the Ts(t,η) in Equation (2).

### 2.2. Extreme Gradient Boosting Evaluator

**Figure 2:**
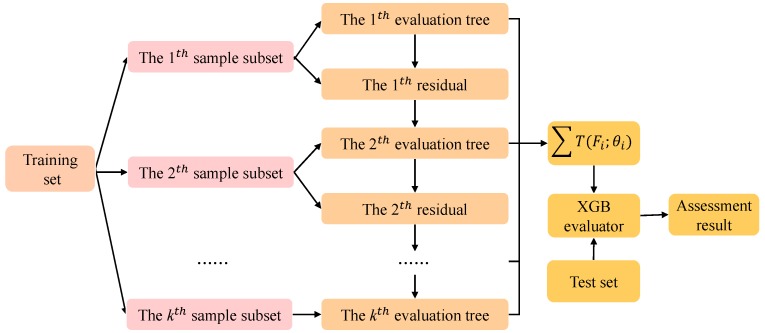
The work flow of the proposed extreme gradient boosting (XGB) estimator.

The proposed extreme gradient boosting (XGB) evaluator utilizes the XGB classification algorithm [31,32] to establish the mapping relationship between features of samples and fault categories in datasets. Specifically, dimensionality reduced data sets are divided into a training set and test set. The main work flow of the proposed XGB estimator is shown in Figure 2.

In Figure 2, the number of integrated evaluation trees is denoted by *k*. For each evaluation tree, a sample subset is constructed by column subsampling [33]. The learning of each evaluation tree is implemented in a serial manner in *k*-round iteration training. For example, the first sample set is put into the XGB algorithm for model training, and the optimal partitioning attribute is selected according to the purity principle at each split node. The evaluation tree grows from top to bottom until all samples in the dataset are evaluated, or all attributes of the nodes are used up.

Based on the residual results, the initial evaluator *f*_1_(*X*) could be fitted as

(4) $$f_{1}(X)=T(F_{1};\theta_{1})$$

where *T*(*F*_1_;*θ*_1_) is the first evaluation tree and *X* represents the training set.

Then, the evaluator *f*_1_(*X*) is used to calculate the residual, denoted by *L*(*y*, *f*_1_(*X*)), where *y* is the label. Additionally, the residual is utilized to fit the second evaluation tree, i.e., *T*(*F*_2_;*θ*_2_). The boosting tree algorithm can realize the performance optimization of the evaluation tree through the forward step-by-step algorithm, thus obtaining the following second-step optimized evaluator *f*_2_(*X*) by

(5) $$f_{2}(X)=f_{1}(X)+T(F_{2};\theta_{2})$$

By further adopting the same step-by-step optimization of the integrated evaluator, a total of *k* evaluation trees *T*(*F_i_*;*θ_i_*) can be generated. Finally, the proposed XGB integrated evaluator is obtained from the combination of these evaluation subtrees:

(6) $$f_{k}(X)=\sum_{i=1}^{k}T(F_{i};\theta_{i})$$

where *F_i_* denotes the sampling subset after column sampling and *θ_i_* denotes the parameter of the *i*th evaluation tree.

XGB adopts a column sampling method to extract sample subsets, thus the correlation between multiple sample sets is low, helping to avoid model overfitting. In addition, the regular term Ω is added to the loss function of the traditional gradient boosting algorithm, and the regularized objective function *L*(Φ) is defined as

(7) $$L(\Phi )=\sum_{i}l({\hat{y}}_{i},y_{i})+\sum_{k}\Omega (f_{k}),\;\mathrm{where}\;\Omega (f)=\gamma N_{T}+\frac{1}{2}\lambda {\|\sigma \|}^{2}$$

in which Φ denotes the samples space, $l({\hat{y}}_{i},y_{i})$ is a differentiable convex loss function, *N_T_* denotes the number of evaluation subtrees, *f_k_* corresponds to the *k*th evaluation tree, σ is the weight of a single tree, and *γ* and *λ* are regular term constants. Generally, the regular term can be regarded as a kind of punishment to the complexity of the model, which helps to smooth the learning weight of the final evaluation model and suppress model overfitting.

In the traditional gradient boosting tree algorithm, the solution of the objective function is approximated in the direction of the negative gradient. To suppress model overfitting and improve the traditional gradient boosting tree algorithm, the second-order Taylor expansion for the loss function is included in the XGB algorithm, namely

(8) $$L^{j}\approx \sum_{i=1}^{n}\left[l(y_{i},{\hat{y}}^{\left(j-1\right)})+g_{i}f_{j}(X_{i})+\frac{1}{2}h_{i}f_{j}{}^{2}(X_{i})\right]+\Omega (f_{j})$$

where *L^j^* represents the simplified regularized objective at the *j*th iteration, *g_i_* represents the gradient direction, *h_i_* denotes the second-order gradient direction, ${\hat{y}}^{\left(j-1\right)}$ is the prediction at the (*j*−1)th iteration, and *X_i_* is the feature vector of the *i*th instance.

By simplifying the objective function and considering the second-order gradient approximation, the model learning efficiency can be effectively enhanced, that is

(9) $${\tilde{L}}^{j}\approx \sum_{i=1}^{n}\left[g_{i}f_{j}(X_{i})+\frac{1}{2}h_{i}f_{j}{}^{2}(X_{i})\right]+\Omega (f_{j})$$

where ${\tilde{L}}^{j}$ represents the simplified regularized objective at the *j*th iteration.

According to Equations (8) and (9), compared with the ordinary gradient boosting integrated evaluator, the proposed XGB evaluator has two outstanding advantages, i.e., a better performance of suppressing model overfitting and a faster model learning rate [31].

## 3. Experimental Setup and Signal Preprocessing

### 3.1. Diesel Engine Rig Test

**Figure 3:**
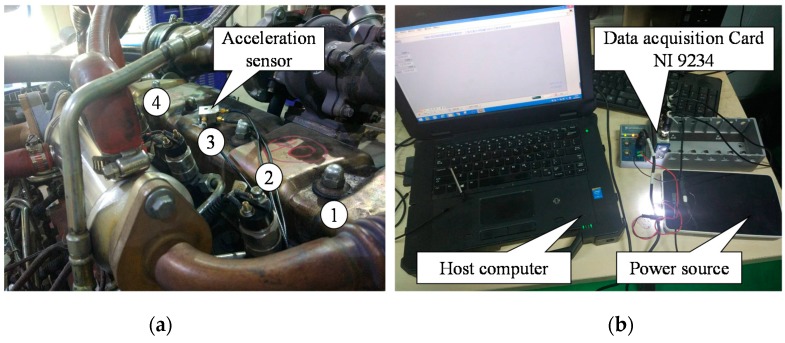
The test rig used for diesel engine misfire assessment: (**a**) Installation of the vibration sensor; (**b**) Data acquisition system.

The experimental setup for diesel engine misfire diagnosis is shown in Figure 3. It is mainly composed of a diesel engine rig, a vibration sensor, and a data acquisition system. The 4A3LR engine used in the experiment was a four-cylinder, four-stroke, intercooled supercharged diesel engine. Considering that its four cylinders were arranged in series, four cylinders were sequentially numbered manually. Meanwhile, the chosen HD-YD-233 vibration sensor was a piezoelectric accelerometer, whose measurable frequency range was 0.5–5000 Hz.

The installation position of the vibration sensor has a crucial influence on whether the acquired vibration signals can accurately reflect the operation status of engine cylinders. Theoretically, to obtain the acceleration signal closest to the actual vibration, the accelerometer should be installed as close as possible to the vibration source of the cylinder. Consequently, to acquire the vibration signal containing as much information as possible, the acceleration sensor was installed at the center of the four-cylinder distribution on the surface of the engine cylinder block. The engine rig was equipped with an FC2000 engine measurement and control system. The main function of the engine measurement and control system is to control the diesel engine so that it works under multiple appointed conditions, so the system was easily utilized to simulate various engine misfire conditions. Moreover, the engine rig was equipped with a vibration buffer bench on the ground floor to reduce the interference caused by the violent vibration of the whole engine bench.

Single-cylinder and double-cylinders misfire are the most common forms of misfire faults. Misfire faults beyond double cylinders will cause severe vibration of the whole engine body, which can be clearly perceived by the operator. Therefore, this paper focused on the single-cylinder misfire and double-cylinders misfire fault types. Specifically, the misfire conditions of the engine running at a low speed (1300 r/min), medium speed (1800 r/min), and high speed (2200 r/min) were tested, respectively. For the single-cylinder misfire fault assessment, five basic working conditions were assigned: normal operation, misfire of first cylinder, misfire of second cylinder, misfire of third cylinder, and misfire of fourth cylinder. In addition, a set of hybrid tests, i.e., normal—single-cylinder misfire—double-cylinder misfire, was added under the speed of 1800 r/min. The specific experimental scheme is shown in Table 1.

### 3.2. Signal Preprocessing

The sampling frequency appointed by the experiment was 25.6 kHz, and the sampling time was 2 min under each working condition. Hence, at least 900 working cycles were included within each sampling time period. A diesel engine is a complex power machine. The coupled vibration of its mechanical structure is generally considered as an inherent noise source, which will interfere with the signal feature extraction process. Therefore, as a key step for signal preprocessing, the signal needs to be denoised. Considering that the wavelet threshold denoising method is especially suitable for non-stationary vibration signals, we employed it to denoise the measured vibration signals, and the crucial parameters are shown in Table 2.

**Figure 4:**
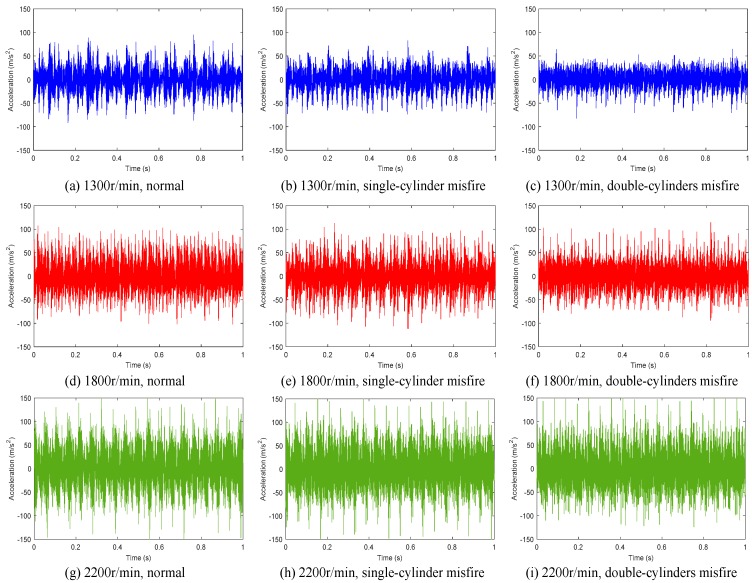
Typical time-domain vibration signal waveforms under different working conditions.

After denoising, a series of typical waveforms were obtained, as shown in Figure 4. It is seen that the vibration severity of the cylinder head shows obvious difference under different speed conditions. Meanwhile, it indicates that when the engine is in different misfire conditions, the time-domain vibration signal shows some differences under the same speed. This demonstrates that the classification and evaluation of misfire faults based on the vibration signal of the cylinder head are feasible, and the key point is to fully exploit the intrinsic characteristics of the signal.

## 4. Experimental Results and Discussion

### 4.1. Feature Extraction

The quality of the signal feature extraction directly affects the evaluation results of the evaluator. To acquire the most sensitive and slightest signal features, the signals were analyzed in the time-domain and hybrid-domain, respectively. Then, related sensitive time-domain and hybrid features could be extracted, thus permitting the construction of a complete feature dataset.

First, to comprehensively characterize the time-domain characteristics of the signals from various aspects, nine statistical features were adopted and extracted in the time domain, i.e., mean value, rectified mean value, root mean square (RMS) value, peak value, peak-to-peak value, kurtosis, shape factor, clearance factor, and margin factor. The calculation of these indexes is presented in Table 3. Among them, the mean value, rectified mean value, and RMS value reflect the overall energy of the vibration signal. The peak value reflects the local strength of the signal, and the peak-to-peak value reflects the degree of signal oscillation. The shape factor, clearance factor, and margin factor detect the impulse components of the signal. Besides, kurtosis reflects the steepness or flatness of the top of the distribution curve. When its value is larger than 3, the distribution is steeper than the normal distribution. Conversely, when the kurtosis is less than 3, the distribution is flatter than the normal distribution.

**Figure 5:**
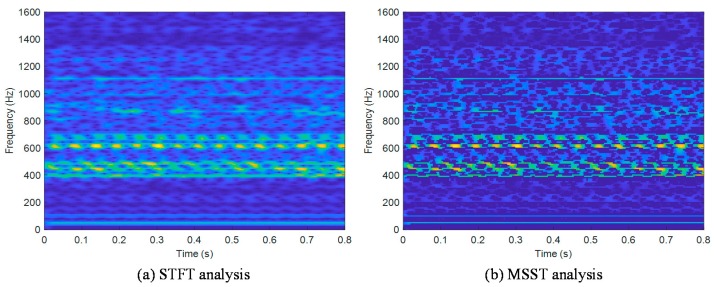
Feature extraction results of frequency bands with two methods.

**Figure 6:**
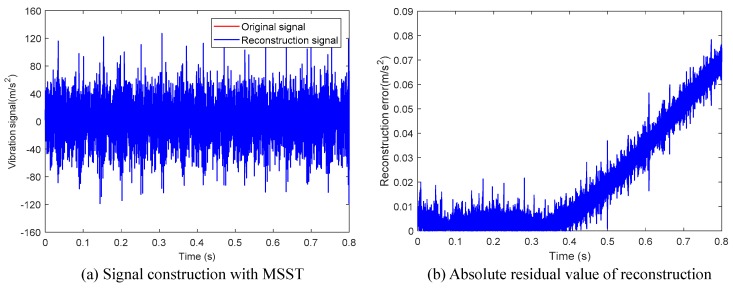
Signal reconstruction and corresponding absolute residual obtained with the multisynchrosqueezing transform (MSST) method.

Then, the STFT method and MSST method were utilized to extract the hybrid domain characteristics of the signal. Taking the vibration signal of the No.2 cylinder at 2200 r/min as an example, the results of these two methods are illustrated in Figure 5. Compared with the STFT method, MSST can effectively remove the high-frequency noise components in the signal, and obviously suppress the diffusion of the characteristic frequency band. Consequently, for several characteristic frequency bands with the greatest energy concentration, the typical characteristic frequency could be obtained. Besides, the signal reconstruction result of the MSST method is presented in Figure 6. It is obvious that the absolute residual value is kept below 0.08 over the entire signal length, which demonstrates that the MSST-based feature extraction method has a stable signal reconstruction capability and does not lose the original characteristics of the signal.

Once the signal is processed by the MSST, each characteristic frequency corresponds to a MSST coefficient. When performing frequency domain feature extraction of a single evaluation sample, the data length *L* is chosen as 3600. Hence, the obtained MSST coefficient *T_x_* is essentially a matrix of 512 × 3600 dimensions; that is, the number *m* of MSST coefficients is 512. It should be noted that the MSST only needs to execute the SST operation once by using a novel algorithm, which can greatly reduce the computational burden [30]. Therefore, the MSST-based feature extraction has a limited computational burden and has potential in real-time diagnosis. In fact, processing data with a length of 3600 only requires a computation time of 0.173 s on a desktop computer (Intel Core i3-6500 3.3 GHz, 4.0 GB of DDR3L RAM, Windows 10 OS). Then, *T_x_* is expanded in the form of a row vector, and the band energy is represented by the sum of the squares of the elements. Specifically, the energy value of the single characteristic band *E_i_* is defined as

(10) $$E_{i}=\sum_{j=1}^{L}{\left(T_{x_{i,j}}\right)}^{2},i=1,2,\cdots ,m$$

where $T_{x_{i,j}}$ denotes the (*i*, *j*)th element of *T_x_*.

On this basis, the m-dimensional column vector R of the characteristic band energy ratio could be obtained, whose element *r_i_* is defined as

(11) $$r_{i}=E_{i}/(\sum_{i=1}^{m}E_{i}),i=1,2,\cdots ,m$$

As mentioned above, the dimensionality of feature vector R is too high. Hence, preliminary simplification is necessary. According to Figure 5, it is seen that the energy of the cylinder head vibration signal is mainly concentrated in the middle and low frequency bands. Therefore, it is considered reasonable to retain the former n-dimensional characteristics. For the five working conditions under the rotational speed of 2200 r/min, we extracted the first 20, 30, 50, and 100dimensional features, and calculated the corresponding energy ratio. The results are shown in Table 4. For simplicity, some abbreviations are used, i.e., W0 denotes the normal condition, and W1–W4 denote the single cylinder misfire conditions. It can be seen from Table 4 that the energy ratios calculated by the top 50 dimensional features under various working conditions basically reached to 98%. On the other hand, if more features are chosen, the signal features are more completely retained (because, if choosing the whole 512 features, the sum of the energy ration would be 100%). However, it is seen that when the 100 dimensional features are selected, the increase of the energy ratios is very limited and the computational cost also increases. To achieve the balance between the accuracy and efficiency, we chose 98% as our baseline and thus utilized the first 50 dimensional features. In fact, by analyzing the classification results for this case, it is found that the classification accuracy using all 512 dimensional features is only slightly higher than that using 50 dimensional features. For working conditions under other rotational speeds, we got similar results. Therefore, the first 50 dimensional features satisfy the accuracy requirements of feature extraction and are thought to be the most appropriate choice when considering both the computational cost and the classification accuracy. Finally, they were used to form the original characteristic data set by combining it with the nine dimensional time-domain features.

### 4.2. Feature Dimensionality Reduction

**Figure 7:**
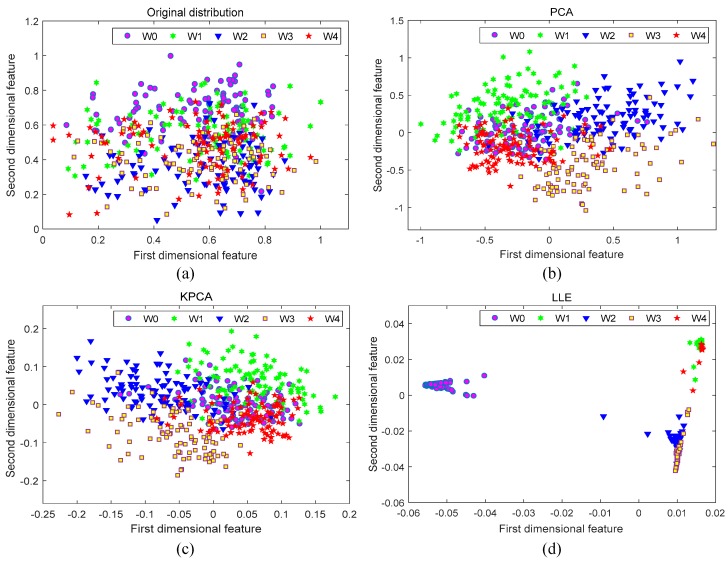
Visualization results of each dimensionality reduction method: (**a**) Original distribution; (**b**) PCA; (**c**) KPCA; (**d**) LLE.

To eliminate the redundant features that affect the generalization performance of the evaluator, further data dimensionality reduction is required. Considering the high dimensionality and nonlinearity of the original characteristic data set, Locally Linear Embedding (LLE) was utilized for further data dimensionality reduction, which is especially expert in processing nonlinear complex data sets [34,35]. The LLE algorithm has a low algorithm complexity and utilizes least squares optimization and matrix diagonalization to obtain highly nonlinear embedding [35]. Since its optimizations are easy to implement and do not require an iterative algorithm, LLE compares favorably in terms of the computation cost compared to purely linear methods, such as Principal Component Analysis (PCA). To begin with, by using the min–max scaling, the feature set was normalized to the unified numerical dimension. To evaluate the performance of the proposed dimensionality reduction method, the preprocessed feature set was processed by LLE, PCA, and Kernel Principal Component Analysis (KPCA). The first two dimensions were calculated separately, and the total sample distribution after dimensionality reduction was observed by data visualization, as shown in Figure 7.

**Figure 8:**
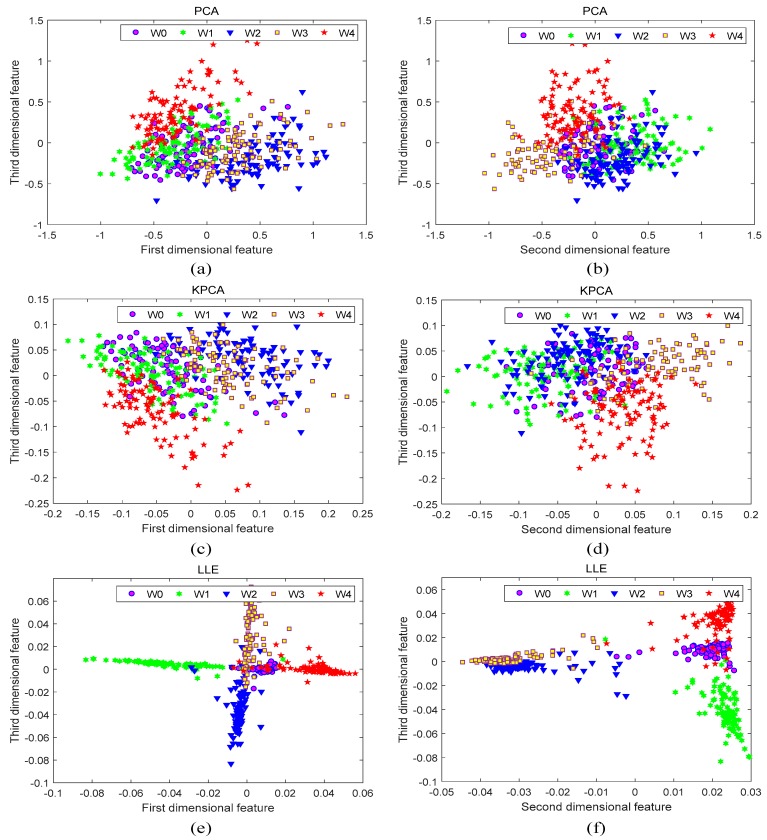
Dimensional reduction effects of other dimensions for Principal Component Analysis (PCA), Kernel Principal Component Analysis (KPCA), and Locally Linear Embedding (LLE): (**a**) The first and the third dimensional features of PCA; (**b**) The second and the third dimensional features of PCA; (**c**) The first and the third dimensional features of KPCA; (**d**) The second and the third dimensional features of KPCA; (**e**) The first and the third dimensional features of LLE; (**f**) The second and the third dimensional features of LLE.

Figure 7a shows the sample distribution of the original feature set. It is seen that the original distribution has the following properties: large intra-class distance, small inter-class distance, and poor linear separability. These unfavorable properties make it difficult to achieve a good classification and evaluation of the samples. Figure 7b–d present the visualization results of these methods. Although the intra-class distance is reduced through the PCA and KPCA dimensionality reduction, the inter-class distance is not obviously improved. The LLE method can not only further reduce the intra-class distance, but also significantly increase the inter-class distance, exhibiting excellent clustering functions. In the first two dimensions, it can be seen from Figure 7d that the LLE can effectively separate W0 from W1, W2, W3, and W4, while W3 and W4 have some overlapping areas. Therefore, the dimensional reduction effects of more dimensions were investigated, and the results are illustrated in Figure 8. It shows that the PCA and the KPCA obtain similar results as the previous case. However, W3 and W4 have been well separated by LLE based on Figure 8e,f. The results of Figure 7 and Figure 8 demonstrate that the LLE method has the best dimensionality reduction effect compared with the other two methods. According to the Ref. [36], the optimal embedding dimension *d* of the LLE algorithm equals the number of fault categories minus 1. Therefore, when applying the rule to this feature set, *d* is taken as 4 in consideration of five types of working conditions.

### 4.3. Results and Analysis

Prior to the specific fault assessment, the misfire fault categories were distinguished by different labels, as shown in Table 5. The whole tests were divided into four groups. Each working condition group contains five types of working conditions. Specifically, the test numbers 1–5, 6–10, 11–15, and 16–20 of Table 1 belonged to the working condition groups I, II, III, and IV, respectively. The former three groups were used to evaluate single-cylinder misfire faults, while group IV was utilized to evaluate single-cylinder and double-cylinder hybrid misfire faults.

**Figure 9:**
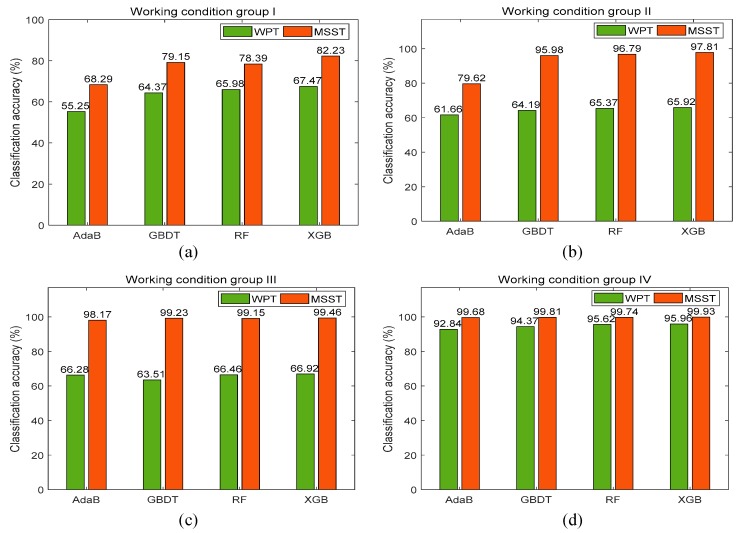
Classification results of the test set under different working conditions: (**a**) Working condition group I; (**b**) Working condition group II; (**c**) Working condition group III; (**d**) Working condition group IV.

Misfire fault diagnosis of a diesel engine is a typical multi-classification problem. For this type of problem, ensemble learning is a suitable solution. According to existing studies, ensemble learning can promote the weak evaluator to a strong evaluator with an excellent generalization performance. Typically, AdaBoost (AdaB), the Gradient Boosting Decision Tree (GBDT), and Random Forest (RF) are the most frequently used ensemble learning algorithms. Moreover, the XGB algorithm improved based on GBDT has sprung up recently and has achieved state-of-the-art results when faced with various pattern recognition problems and competitions. Therefore, the XGB algorithm has been employed to implement accurate fault classification of diesel engine misfire, which can effectively avoid model overfitting. To verify the proposed MSST-based feature extraction method, wavelet packet transform (WPT) was utilized to extract the wavelet packet features. On this basis, by combining the MSST features and WPT features with the above four recognition methods, comparisons among eight different evaluation methods were made to verify the performance of the proposed method. For each working condition group, sixty percent of the total samples were selected to construct the training set, and the rest were chosen as the test set. The evaluator was trained on the training set, and the test was performed on the test set to obtain the classification accuracy. In order to make the classification results more reliable, each method was repeated 20 times to obtain the average diagnosis accuracy, and the classification results of four groups are shown in Figure 9.

According to the classification results obtained with these recognition methods, the proposed MSST-XGB evaluation method achieved the highest classification accuracy for all working conditions. For working condition group IV, the highest classification accuracy of the proposed method was up to 99.93%. It can be seen that for the same recognition method, the classification accuracy of the evaluator trained by the MSST-based features is nearly 24.63% higher on average than that of the evaluator trained with the WPT features. Therefore, it verifies the superiority of the MSST-based feature extraction. Meanwhile, the classification accuracy of the proposed method achieves about 11.23%, 1.75%, and 1.79% higher on average than that of AdaB, GBDT, and RF with the same MSST-based feature extraction. In summary, the proposed MSST-XGB evaluation method can achieve a high classification accuracy for the fault diagnosis of diesel engine misfire.

**Figure 10:**
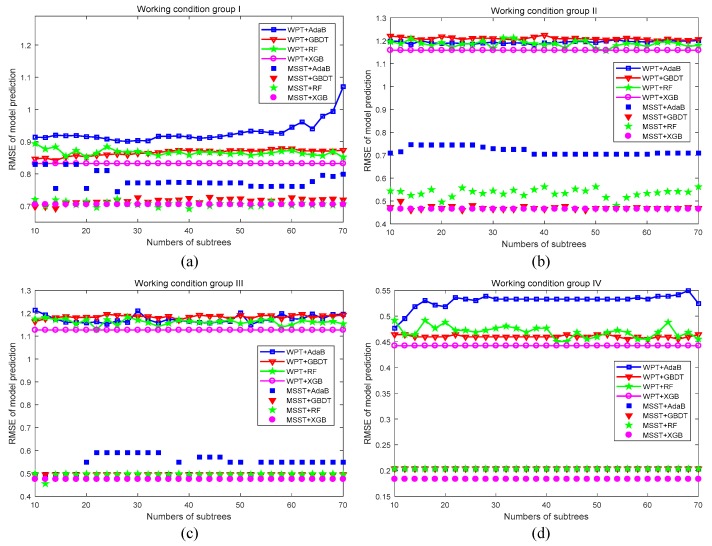
Root mean squared error (RMSE) values obtained under different evaluation approaches: (**a**) Working condition group I; (**b**) Working condition group II; (**c**) Working condition group III; (**d**) Working condition group IV.

Meanwhile, in order to verify the robustness of the proposed evaluation method, the relationship between the model prediction root mean squared error (RMSE) and the number of evaluation subtrees was investigated. The corresponding RMSE curve results are shown in Figure 10. The RMSE describes the average evaluation accuracy, while the number of subtrees reflects the complexity of the model. In Figure 10a, for instance, the magenta solid line with a hollow circle characterizes the trend of the RMSE of the WPT-XBG evaluation method with the number of subtrees. Compared with the WPT-AdaB evaluation method, i.e., the blue solid line with a hollow square in Figure 10a, the WPT-XBG evaluation method achieves smaller RMSE with the same complexity of the model, i.e., number of subtrees. Therefore, Figure 10 reflects the relationship between the average evaluation accuracy and model complexity. It is seen from Figure 10 that the RMSE values of the four types of evaluation methods using MSST-based feature extraction are much smaller than the same recognition methods using WPT-based feature extraction, which proves that evaluation methods using MSST-based features can achieve a more stable evaluation performance. Meanwhile, Figure 10 also shows that the evaluation accuracy of XGB and RF is much better than that of AdaB and GBDT. It should be noted that when the model complexity is high enough, the evaluation robustness of RF is slightly better than XGB with the WPT-based feature extraction. However, this is at the cost of model over-fitting, making it difficult to guarantee the comprehensive performance for the misfire diagnosis of a diesel engine. According to Figure 10, the proposed MSST-XGB evaluation method for misfire fault diagnosis could obtain the minimum RMSE value with minimum number of evaluation subtrees. It demonstrates that the proposed method can ensure the most stable evaluation performance under the minimum model complexity compared with other methods, and can also effectively prevent the model from overfitting. In conclusion, the proposed evaluation method has the best generalization performance and robustness and an excellent ability to suppress the model overfitting.

## 5. Conclusions

In this study, a novel fault diagnosis framework with a high accuracy and strong robustness for diesel engine misfire has been presented. To overcome the intrinsic weakness of the obtained characteristic low resolution with traditional time–frequency analysis approaches, a hybrid feature extraction method that combines time-domain statistical characteristic parameters and MSST-based features has been proposed to acquire the most sensitive and slightest signal features. To address the problem of nonlinearity and information redundancy of the raw data set, a dimensionality reduction algorithm based on the LLE algorithm has been applied. Finally, due to the excellent generalization performance and the superior ability of suppressing model overfitting, the XGB classification algorithm has been utilized to construct an appropriate ensemble evaluator to finish the task of misfire fault classification.

Various misfire fault tests have been carried out on a test rig of a diesel engine to verify the effectiveness of the proposed MSST-XGB evaluation method. Four types of pattern recognition methods (AdaB, GBDT, RF, and XGB) have been combined with WPT-based and MSST-based feature extraction methods to construct eight evaluation approaches. The results demonstrate that the proposed MSST-XGB evaluation method achieves the highest classification accuracy for all working conditions, and the highest classification accuracy of the proposed method on the test dataset was up to 99.93%. Meanwhile, the classification accuracy of the evaluator based on the MSST-based feature extraction is approximately 24.63% higher on average than those evaluation approaches using WPT-based feature extraction. Moreover, the proposed evaluation approach obtains an excellent robust performance and has a superior ability to prevent model overfitting. In conclusion, the proposed method achieves the best overall performance for the fault diagnosis of diesel engine misfire.

## Figures and Tables

**Table 1:** List of test conditions.

Serial Number	Rotating Speed n (r/min)	Working Condition
1	1300	Normal operation
2	1300	Misfire of first cylinder
3	1300	Misfire of second cylinder
4	1300	Misfire of third cylinder
5	1300	Misfire of fourth cylinder
6	1800	Normal operation
7	1800	Misfire of first cylinder
8	1800	Misfire of second cylinder
9	1800	Misfire of third cylinder
10	1800	Misfire of fourth cylinder
11	2200	Normal operation
12	2200	Misfire of first cylinder
13	2200	Misfire of second cylinder
14	2200	Misfire of third cylinder
15	2200	Misfire of fourth cylinder
16	1800	Normal operation
17	1800	Misfire of second cylinder
18	1800	Misfire of second and third cylinders
19	1800	Misfire of second and fourth cylinders
20	1800	Misfire of first and second cylinders

**Table 2:** Crucial parameters of the wavelet threshold denoising function.

Wavelet Basis	Threshold Function	Threshold Processing Criteria	Decomposition Layer
db4	Soft threshold	Unbiased likelihood estimation	4

**Table 3:** Definition of utilized statistical features in the time-domain for the vibration signal.

Time-Domain Feature	Equation	Time-Domain Feature	Equation
1. Mean	$\bar{x}=\frac{1}{L}\sum_{i=1}^{L}x_{i}$	6. Kurtosis	$x_{kur}=\frac{1}{L}{\sum_{i=1}^{L}\left(\frac{x_{i}-\bar{x}}{\sqrt{\frac{1}{L}\sum_{i=1}^{L-1}{\left(x_{i}-\bar{x}\right)}^{2}}}\right)}^{4}$
2. Rectified mean	${\bar{x}}_{abs}=\frac{1}{L}\sum_{i=1}^{L}\left|x_{i}\right|$	7. Shape factor	$x_{sf}=\frac{x_{rms}}{\frac{1}{L}\sum_{i=1}^{L}\left|x_{i}\right|}$
3. RMS	$x_{rms}=\sqrt{\frac{1}{L}\sum_{i=1}^{L}x_{i}{}^{2}}$	8. Clearance factor	$x_{clf}=x_{pp}/x_{rms}$
4. Peak	$x_{pk}=\max(|x_{i}|)$	9. Margin factor	$x_{mf}=x_{pp}/{\left[\frac{1}{L}\sum_{i=1}^{L}\sqrt{\left|x_{i}\right|}\right]}^{2}$
5. Peak-to-peak	$x_{pp}=\max(x_{i})-\min(xi)$		

**Table 4:** The energy ratio of extracted feature dimensionalities.

Dimensionality	Signal Energy Ratio of Top n Features (%)				
	W0	W1	W2	W3	W4
20	90.89	93.64	91.35	92.32	93.28
30	95.34	96.23	95.77	95.89	96.13
50	98.97	99.08	98.47	98.95	98.84
100	99.05	99.10	98.74	99.02	98.91

**Table 5:** Label lists of working conditions.

Group I, II, and III		Group IV	
Working Condition	Label	Working Condition	Label
Normal	1	Normal	1
No.1 cylinder misfire	2	No.2 cylinder misfire	2
No.2 cylinder misfire	3	No.2 and No.3 cylinder misfire	3
No.3 cylinder misfire	4	No.2 and No.4 cylinder misfire	4
No.4 cylinder misfire	5	No.1 and No.2 cylinder misfire	5

## Data Availability

The data sets and codes used in this paper to support the findings of this study are available from the corresponding author upon request.
